# Path Generator with Unpaired Samples Employing Generative Adversarial Networks

**DOI:** 10.3390/s22239411

**Published:** 2022-12-02

**Authors:** Javier Maldonado-Romo, Alberto Maldonado-Romo, Mario Aldape-Pérez

**Affiliations:** 1 Institute of Advanced Materials and Sustainable Manufacturing, Tecnologico de Monterrey, Mexico City 14380, Mexico; 2 Centro de Innovación y Desarrollo Tecnológico en Cómputo, Instituto Politécnico Nacional, Unidad Profesional Adolfo López Mateos, Juan de Dios Bátiz s/n esq. Miguel Othón de Mendizábal, Mexico City 07700, Mexico; 3 Centro de Investigación en Computación, Instituto Politécnico Nacional, Unidad Profesional Adolfo López Mateos, Juan de Dios Bátiz s/n esq. Miguel Othón de Mendizábal, Mexico City 07700, Mexico

**Keywords:** neural networks, machine learning, unpaired datasets, path generator

## Abstract

Interactive technologies such as augmented reality have grown in popularity, but specialized sensors and high computer power must be used to perceive and analyze the environment in order to obtain an immersive experience in real time. However, these kinds of implementations have high costs. On the other hand, machine learning has helped create alternative solutions for reducing costs, but it is limited to particular solutions because the creation of datasets is complicated. Due to this problem, this work suggests an alternate strategy for dealing with limited information: unpaired samples from known and unknown surroundings are used to generate a path on embedded devices, such as smartphones, in real time. This strategy creates a path that avoids virtual elements through physical objects. The authors suggest an architecture for creating a path using imperfect knowledge. Additionally, an augmented reality experience is used to describe the generated path, and some users tested the proposal to evaluate the performance. Finally, the primary contribution is the approximation of a path produced from a known environment by using an unpaired dataset.

## 1. Introduction

Different academic domains, as well as daily life, have been incredibly touched by interactive multimedia systems [1]. For instance, they provide capabilities for robotic exploration in order to locate obstacles in a physical area [2]. In the realm of e-learning, materials are displayed to help students understand abstract concepts [3]. The use of virtual elements in systems for training in the healthcare industry was described in [4,5]. Therefore, the primary contribution of an interactive multimedia system is the enhancement of real-world settings with virtual components. Such technologies are described by the concepts of augmented reality (AR) and mixed reality (MR). AR technology allows computer-generated virtual visuals to perfectly overlay real-world objects in real time [6]. MR, on the other hand, enables concurrent work in both the physical and virtual domains, hence minimizing domain transition costs [7]. The primary distinction between MR and AR is that MR provides a more immersive experience.

Since MR requires a comprehension of the surroundings, specific equipment that contains sensors to sense the environment is required [8]. In order to provide a real-time user-interaction experience, the system must handle a great volume of data, which generates numerous important opportunities and difficulties [9]. Consequently, the current equipment is intended for a limited audience due primarily to its technological qualities, which limit the gradual extension of this experience on a large scale [10]. Nevertheless, unlike in MR, an immersive experience in AR is unnecessary, which reduces the prices and requirements for equipment features. For instance, mobile devices, such as smartphones, are an integral part of people’s everyday lives, generating a vast presence in conducting a variety of tasks. In 2020, there were at least 50 billion gadgets [11]. These gadgets have provided diverse interactive experiences, including AR experiences, mostly through markers and telemetry [12].

Existing technologies that enable interactive AR experiences, such as the ARCore and ARKit frameworks, make it possible to comprehend interactions with the environment in real time [13]. These frameworks concentrate on gathering information from flat surfaces to display virtual elements, but these frameworks disregard the fact that items may produce an accident between the user and the surrounding environment [14,15]. Therefore, current solutions lack an approach to this fundamental obstacle when providing this type of application: an environment’s security. The authors of [16] defined a collision as the avoidance of a path or trajectory between two configurations embedded in the cost field while motion restrictions are considered. Similarly, free space allows agents to interact freely with the environment to avoid accidents.

For this reason, environmental comprehension is essential to the real-time processing of the environment. In order to apply solutions that enhance the features of constrained equipment, data processing that employs machine learning (ML) techniques is required [17]. For instance, the authors of [18] emulated the behavior of a sophisticated sensor by using machine learning. Deep convolutional neural networks (DCNNs) reduce the number of operations and can operate on devices with limited computational resources [19]. In addition, this technique was supplemented by generative adversarial networks (GANs) to create depth image samples [20]. These enabled the production of a depth image via competition between two networks.

The creation of a specific dataset is a significant constraint of existing methods. Such datasets comprise paired samples, since the predicted sample and source image have linked attributes. This constraint creates a disadvantage for this type of dataset in terms of data collection. For example, RGB-D data are required in order to perceive the scene, but this strategy could be more effective in undetermined scenes, since depth information is necessary to estimate samples. Since the predicted images are unconnected to the source images, this problem involves unpaired samples [21]. However, the concept of the neural transfer style, which involves learning the features from one sample to another, is an alternative method for merging sample features [22]. Cycle-GAN and pix2pix are two concepts for combining the properties of one domain with those of another [23]. In order to interact with a known environment without prior information, an unknown domain modifies its transfer style.

As a result of the above, this proposal offers a path planner generator for avoiding collisions by employing GANs. In addition, the architecture purports to be a substitute for interacting with numerous settings in order to minimize design time and enable the use of smartphones and other devices with few resources. Consequently, the following hypothesis is investigated in this study. Since the GAN permits the transfer of samples from one domain to another, it could infer the properties between domains to connect samples from a known situation with paths through an unfamiliar environment.

This work investigates and explains the influence of inferring features from a known scenario to an unknown scenario in order to approximate a generic solution for creating pathways in real time on low-cost devices.

The remaining sections of this manuscript are structured as follows. Section 2 describes the context and research gaps. The suggested work is introduced in Section 3. Section 4 illustrates the outcomes and analyses of the experiments. The conclusion is then stated in Section 5.

## 2. Background and Research Gaps

In the research field of autonomous intelligent systems, path planning is a problem where the system determines the optimum collision-free path between two points. Usually, the implemented architecture incorporates the perception and the planning modules separately, forming a complex architecture to offer a real-time performance [24,25]. Nevertheless, the performance in the real world is inefficient in terms of time and resources [26]. As a result, virtual simulators expedite the design process. Simulators such as Airsim help evaluate agents’ dual systems [27]. Therefore, simulators generate samples of possible scenarios for training algorithms that require real-time perception based on ML techniques [28].

**Figure 1:**
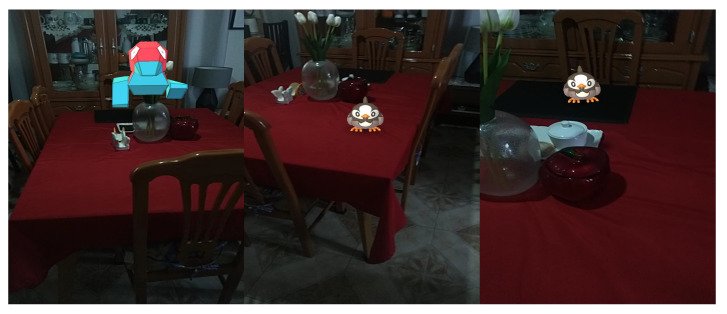
Displays of virtual elements on flat surfaces using the Pokemon Go game. The virtual elements are displayed through obstacles within the scenario that could cause accidents because the user requires interaction with the virtual objects.

The perception of the environment requires specialized sensors, but the current tools require previous interactions to be effective. For instance, ARcore needs to move the device to detect key features to build a 3D environment described by the ORB-SLAM approach [29]. Once the system scans the scene, flat surfaces are detected, offering real-time interaction with the scenery, but the system positions virtual elements in places that are difficult to access or that may cause accidents. For example, Figure 1 shows a virtual element on a flat surface, offering a limited experience in terms of interaction with the environment because the selection of the surface is random, generating some inconvenience in the experience. However, mobile devices require high computer performance but are limited in computer power and battery. Therefore, AR and MR experiences require understanding the environment to determine the best place to display the virtual elements.

Since specific sensors are required and avoid previous interaction with the environment, different proposals have offered alternative solutions, such as replacing the sensor with ML algorithms [30]. Existing evidence where ML replaces specialized smartphone sensors, as described in [31], is used to accurately replace a depth sensor from an image taken from the device. Likewise, another alternative solution is to employ simulators and ML approaches to connect a sample-limited environment with a known environment [32]. Thus, ML techniques provide novel features to the current systems.

On the other hand, the high computing power on an embedded device is high in time and resource needs, limiting the roll-out on generic devices. Therefore, the end-to-end approach reduces external elements such as sensors since the perception of the scenario is considered within a simulation [33]. This approach reduces the conventional architecture and additional sensors to generate the necessary information to perceive the environment.

Although considerable efforts have been made to create alternatives through ML techniques, there is a severe issue where the current solutions focus on particular problems. In other words, a dataset is required for each environment. Since the current solutions implement datasets in the training environment based on specific environments, we have detected that GANs have been used to perform feature blending between unknown domains through images [34,35]. Therefore, GANs can offer novel solutions by exploring domain changing through style transfer between two domains.

**Figure 2:**
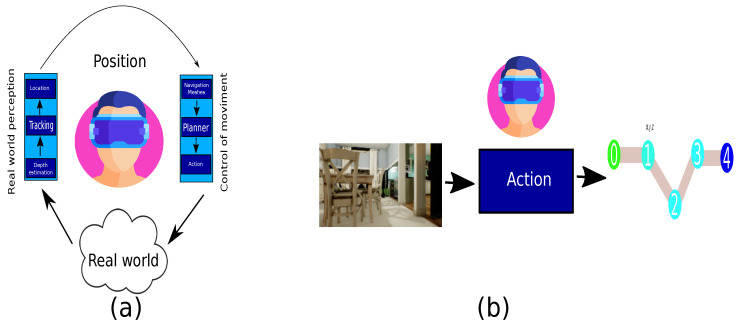
Path generator architectures. (**a**) Conventional architecture. (**b**) Reduced architecture.

The current work aims to contribute to the analysis of the behavior in feature inference between an image acquired from an unknown domain and translate it to a controlled scenario to generate a safe path and evaluate its behavior and determine the reliability of deploying virtual elements in augmented reality applications in real time. Furthermore, the conventional architecture described by Figure 2a is composed of two main modules, the perception and the planner. Likewise, the conventional architecture is reduced for being employed on embedded devices only utilizing a single module, as Figure 2b illustrates.

## 3. Proposed Work

**Figure 3:**
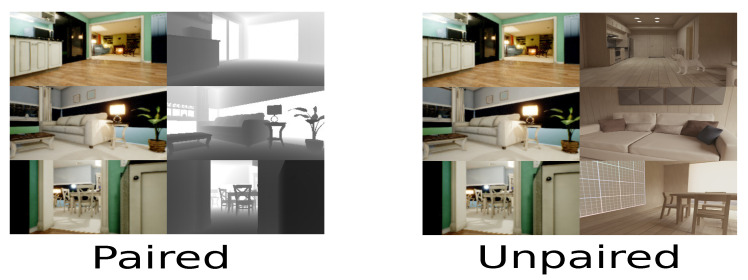
Type of samples for the known paired domain and the unknown domain unpaired. The paired samples have a 2D image and depth data to calculate the occupied obstacles. On the other hand, unpaired samples have two principal samples for indistinct environments.

The present proposal takes the principal features of cycle-GAN and pix2pix models since these approaches generate a new sample according to features of an unknown domain. Based on this approach, the first step is to define the available domain to generate the dataset with known paths connected to the unknown environment. The referent environment is known because it includes the depth data for each sample. In contrast, the depth value in the second scenario is unknown because the depth data is unavailable. As depicted in Figure 3, the virtual simulator generates paired samples, and the samples of the unknown scenario are unpaired because the relation between data is unfamiliar.

**Figure 4:**
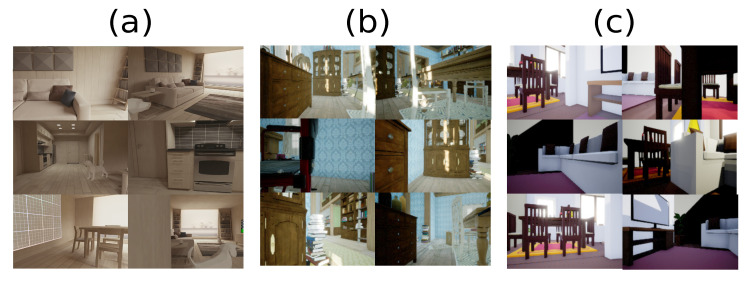
Unknown environments with different features, such as illumination, texture, and color. However, the environments involve furniture as a principal element. (**a**) It is an Android environment emulator, and the unreal store downloaded (**b**,**c**).

In order to describe the performance of the experiment, four environments were defined. An environment represents a domain since a domain contains elements with similar features. Therefore, each environment has different features, such as illumination, color distribution, and textures, as shown in Figure 4. The following analysis describes the performance for each domain according to features such as the average color and dominant color employing the k-means approach [36]. These outcomes are described in Table 1. It is observed that it is challenging to determine whether transfer and regular features exists in relation with the known environment because the unknown environment *A* has a bad behavior since the dominant color tends to be black. Consequently, the interpretation of data is limited to determining the relation of approximation to the expected environment.

**Figure 5:**
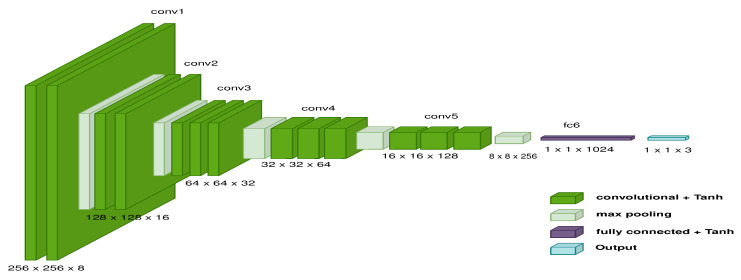
Features of the DCNN model to describe samples in a 3D space.

**Figure 6:**
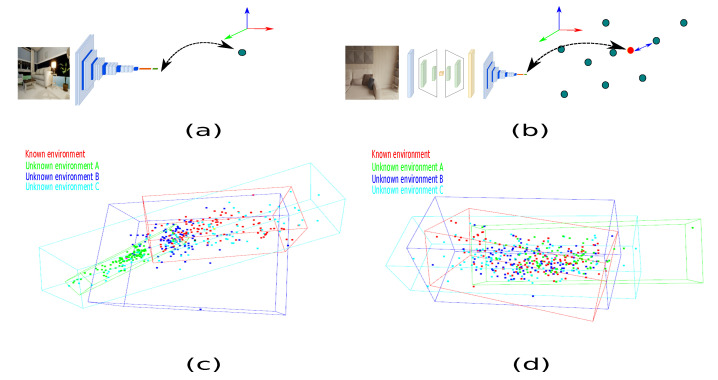
Samples plotted in a 3D space. (**a**) A DCNN plots a sample in 3D space. (**b**) Implementation of the transfer style approach. (**c**) Three-dimensional description of the distribution of samples between different domains. (**d**) Graphical description of the samples with transfer style.

In order to describe the data, each image is plotted in a three-dimensional space. Therefore, the sample is converted into a 3D point. This analysis employs a deep convolutional neuronal network (DCNN) model to plot a sample in 3D space; Figure 5 describes the characteristics. For this case, the model initializes the variables arbitrarily. Consequently, the distribution of the elements in space varies based on the random values, and Tanh has been implemented as a transfer function because the data are distributed throughout several spatial regions (Figure 6a). According to Figure 6b, the transfer style approximates the unknown sample to a known sample. This behavior shows that the GAN trough transfer style positively affects the data between two domains.

Due to the features of each domain depicted in Figure 6c, the unknown domains are distributed throughout the 3D space. Red, for instance, indicates a recognized environment, while green, blue, and cyan represent unknown environments. As is shown in Figure 6d, the approximation from unknown domains to known domains is near since the data concentration covers a similar space region after applying the GAN architecture. Similarly, the centroid of each domain and its distance from the known environment are determined. According to Table 2, the centroid for each domain is close to the known environment centroid. Therefore, the GAN approximates the characteristics between unidentified space domains.

**Figure 7:**
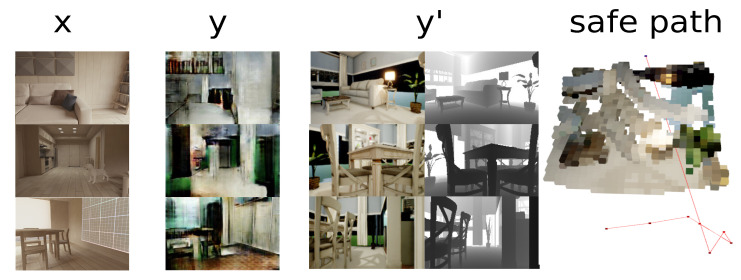
Change in domains to achieve a path in a known scenario. In the first stage (X), the sample from the unknown domain is translated to the known domain (Y). Subsequently, the samples from the known domain are approximated to generate the path (Y’).

This proposal adds a step to the GAN architecture once the features have been approximated: a hierarchical clustering (HC) [37] where the closest sample is obtained based on the distance between the generated sample and the known samples. Since the accessible domain contains depth information for determining a path, the generated sample must approximate a known sample. Furthermore, the rapidly exploring random tree (RRT) algorithm establishes the collision-free path [38], as is shown in Figure 7.

To generate a path, the calculus of variations describes the composition of a path [39] as the sum of the distances between two consecutive places defined by Equations (1) and (2). Consequently, a path consists of a collection of lines in space. Likewise, one characteristic of a path is its capacity to avoid obstacles. Path planning is the shortest distance between *m* obstacles *O* and the best value with the highest level of free collisions in a series of points *p* of length *n*. In this sense, the maximum optimization problem is changed into the minimum optimization problem by adding a negative sign to the value, as stated by Equation (3) [40].

(1) $$distance\left(p_{i},p_{i+1}\right)=\sqrt{{\left(x_{i}-x_{i+1}\right)}^{2}+{\left(y_{i}-y_{i+1}\right)}^{2}}$$

(2) $$length\left(p\right)=\sum_{i=0}^{n}distance\left(p_{i},p_{i+1}\right)$$

(3) $$path\left(p\right)=-\min\left\{minDistance(p_{i}p_{i+1},O_{j})\right\}$$

**Figure 8:**
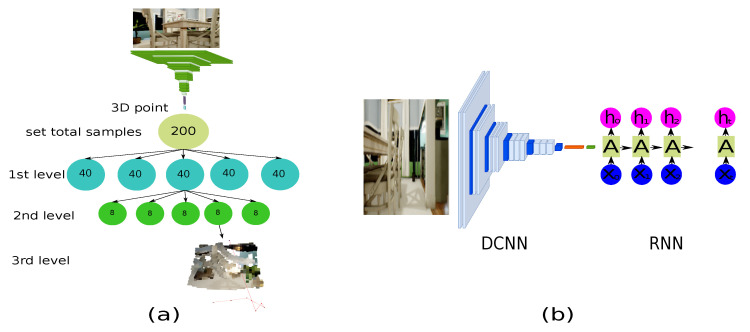
Path generator approaches. (**a**) A hierarchical cluster is composed of the centroid of each set to avoid analyzing whole samples. (**b**) Autoencoder comprises a deep convolutional neuronal network as the encoder and a recurrent neuronal network as the decoder.

The following step is to develop path generators based on ML using an end-to-end approach. Regarding this study, [41], we have presented two methods for developing a path-planning generator. The first method analyzes the input sample based on the close distance between the centroid and each cluster level until a sample with the minimum distance is obtained, as shown in Figure 8a). Figure 8b) describes the second approach, which is an autoencoder composed of two types of networks: a DCNN and a recurrent neural network (RNN).

**Figure 9:**
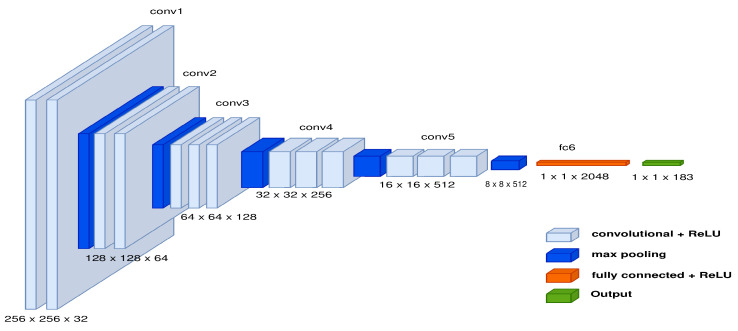
Features of the DCNN model to extract characteristic vector.

Since the second method is an autoencoder, the encoder made of DCNN has the features shown in Figure 9 to extract a characteristic vector, and the RNN is responsible for producing a series of points in 3D space. Moreover, a vocabulary is necessary because it was inspired by the picture caption algorithm [42]. The vocabulary consists of discrete samples of the three-dimensional space, and the 2-meter space is split into 20 cm for each step. As a result, the vocabulary decreases the number of discrete-sized samples on each side. Once discrete samples have been gathered, each trajectory calculates the frequency of each node. In this instance, at least 1000 potential values were reduced to 183. Consider that the total number of samples depends on the size of the discrete sample and that the samples generated vary based on the RRT algorithm’s generated pathways.

**Figure 10:**
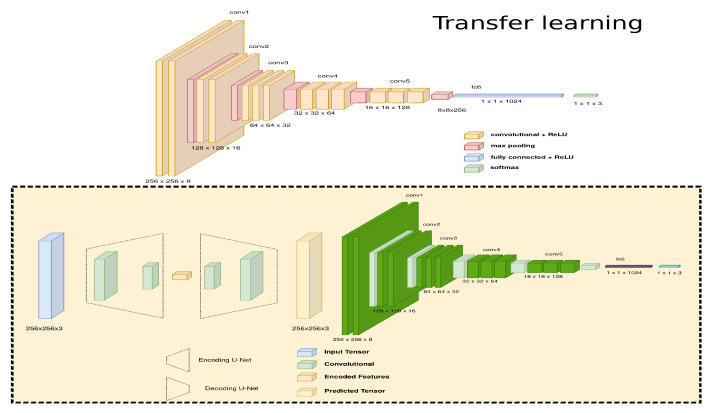
Transfer learning reduces the number of operations since a complex problem with many features is replaced by a model with a smaller number of features.

According to [43], a real-time system generates at least ten frames per second. In order to offer a real-time experience, the architecture must be simplified to be deployed on a mobile device. The transfer learning approach replaces a complex design with fewer features to solve the same problem [44]. As demonstrated in Figure 10, the GAN architecture with HC is simplified to a DCNN since a smartphone can execute this type of model without any difficulty.

**Figure 11:**
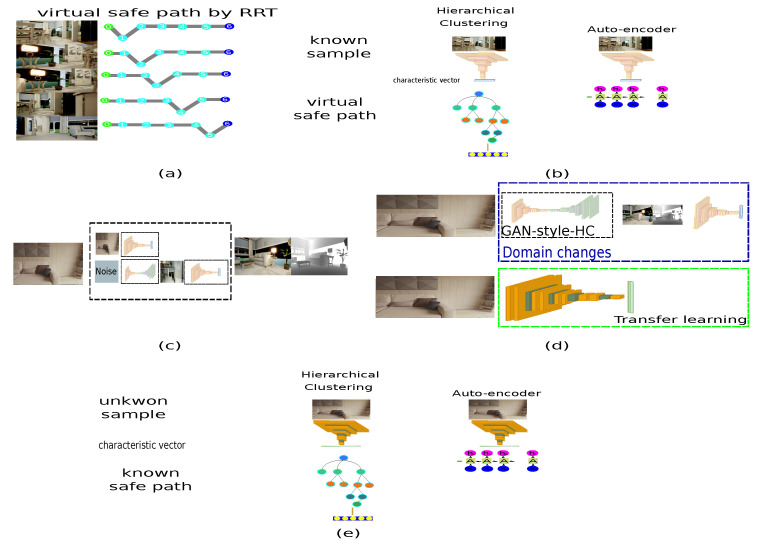
Proposed architecture to generate a path in augmented reality applications on embedded devices employing unpaired samples. (**a**) Virtual path associated with each virtual sample. (**b**) Three machine learning approaches to generate a virtual path. (**c**) Generative adversarial network to connect unknown samples with the known environment. (**d**) Transfer learning to reduce the architecture to employ embedded devices. (**e**) The connection between unknown samples with a virtual environment generates a path with free collisions.

The methodology is summarized in Figure 11, which depicts the implementation of a lengthy process in an embedded device using unpaired samples from two environments. The paths provided by the RRT algorithm in a known environment are described in Figure 11a. Figure 11b depicts how the following step constructs the path using HC and autoencoder. Likewise, Figure 11c uses the GAN style generator to convert the characteristics across two domains employing the HC approach to approximate a known path. In order to use the design on embedded devices, Figure 11d implements the transfer learning approach, which describes the 200 samples of the optimized architecture saved in an unknown environment. Figure 11e illustrates the relationship between an unfamiliar and known path for unpaired samples. In addition, an augmented reality system for smartphones is proposed to display the behavior of the development of paths, which generates a path in real time.

The performance of this proposal is evaluated based on the behavior in creating an expected vector by a machine learning model and the collision-free coefficient that characterizes the viability of the generated path.

## 4. Experimental Phase and Analysis

The proposed architecture was implemented in a g4dn.xlarge instance in Amazon Web Services (AWS) with the following specifications: 4 VCPU XEON 8259CL 2.5 GHz, 16 GB RAM, 125 GB SDD storage, with NVIDIA Tesla T4 GPU with 320 Tensor Core with 16 GB RAM. The introduced architecture was implemented in Tensorflow 2.4. Training time was 1 h and 10 min. The implementation on the smartphone employs Tensorflow-lite. Along with the experimental phase, 300 samples were used for the GAN architecture; finally, 50 unknown samples were used for the experiment. There are three distinct categories of physical items in the surrounding area. Two of these things are chairs of various colors and sizes, while the last object is a table. Microsoft Kinect V1 has a minimum perception range of 40 cm when configured for a 4 m range [45]. Therefore, things must be larger than 40 cm to be perceived at a range of up to 4 m. Consequently, we have both flat and curved surfaces to measure data consistently.

The RRT algorithm returns a vector representing each sample’s path. The following experiment compares the autoencoder’s and HC’s performance between known and created vectors. The behavior is based on the Euclidean distance (Equation (4)), the Manhattan distance (Equation (5)), and the cosine similarity (Equation (6)) used to examine the difference between the predicted vector *x* and the generated vector *y*. The free collision coefficient of Equation (7) quantifies whether or not at least one node generates an inadequate path.

(4) $$euclidean=\sqrt{\sum_{i=0}^{k}{\left(\vec{x_{i}}-\vec{y_{i}}\right)}^{2}}$$

(5) $$manhattan=\sum_{i=0}^{k}\left|(\vec{x_{i}}-\vec{y_{i}})\right|$$

(6) $$cosine\;similarity=\frac{\vec{x}\cdot \vec{y}}{∥\vec{x}∥∥\vec{y}∥}$$

(7) $$C_{free\;collision}=1-\frac{\sum_{i=1}^{N_{samples}}\left\{\begin{array}{c} if\;exists\;collision\;c=1\\ else\;c=0 \end{array}\right.}{N_{samples}}$$

The experiment assesses the behavior in four contexts to develop a path. Furthermore, the suggested method consists of the original model and the transfer learning method, with 50 samples for each model. Comparing the created and predicted vectors, Table 3 illustrates the behavior of path generation. A proposed collision-free coefficient describes the behavior because the coefficient decreases when a possible collision is likely in the path. In addition, distance-measuring devices such as ARcore and the Kinect sensor have been used as references. It is crucial to mention that ARcore and Kinect sensor measurements were only conducted once.

According to the statistics, the model with the best performance is the HC with transfer learning. Given the HC approach’s characteristics, it approximates a near-optimal solution because the training samples are sufficient for the scenario features. However, this algorithm lacks efficiency because more samples are required when the scenario expands. On the other hand, it has been observed that the use of TL reduces characteristic vector resolution for each sample because the vector is normalized, and the word size change directly influences path generation. The autoencoder with TL exhibits this behavior because a fluctuation in the model’s word size is another value that causes the error to grow; an alternative option is to increase the number of samples when performing inference in the TL model. ARcore and Kinect sensor offers the best performance, but integrating these technologies is challenging because a path must be established online and requires additional considerations, for example, previous environment exploration for ARcore and located objects in at least 40cm for the Kinect sensor.

**Figure 12:**
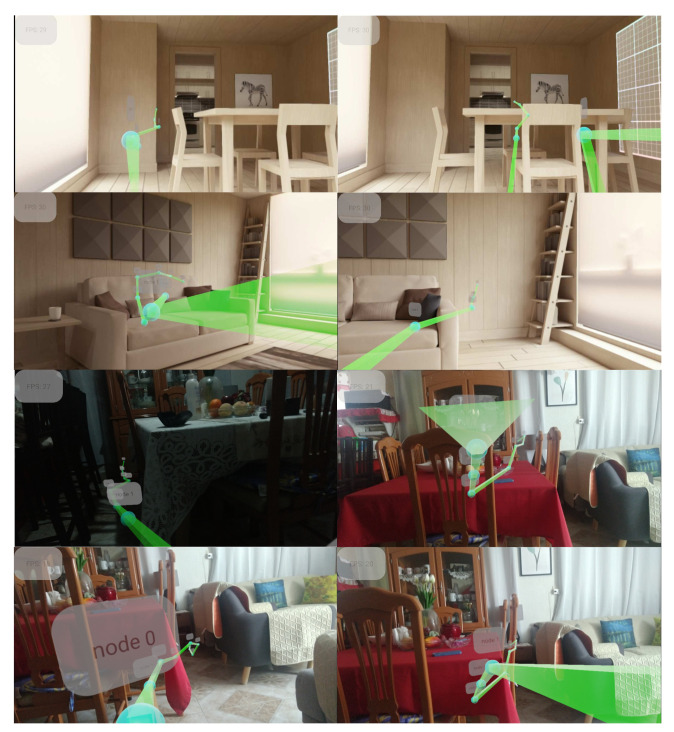
The path generated for augmented reality experiences in real-time. A line indicates the path to display virtual elements to avoid physical objects.

Since HC has the best performance in this experiment, it has been implemented on a Moto X4 smartphone with a Qualcomm 630 processor. In addition, the proposal employs the PointCloud function of ARCore to determine when the input data are updated. Thus, the path is formed when the features of the current image are relevant to the new image, obtaining a maximum sampling rate of 30 frames on the test device. Figure 12 illustrates a path that depicts a 2-meter-long path devoid of collisions. Observe that a route is provided to avoid obstacles in the environment. The test evaluation was conducted in two unknown environments using an AR tool. The path avoids physical elements, implementing a domain change with a transfer style.

**Figure 13:**
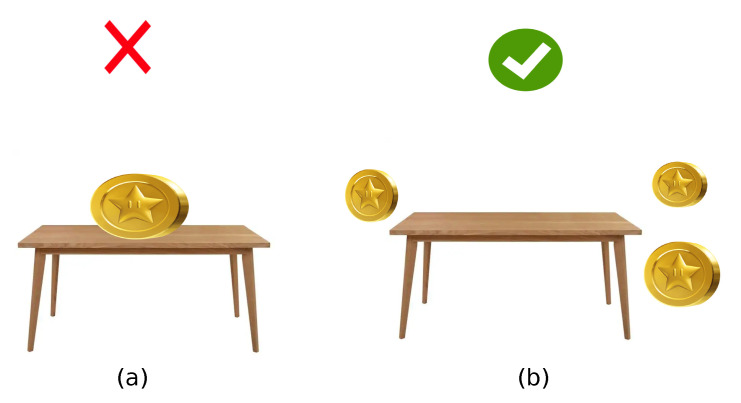
Location of virtual elements. (**a**) Unsafe location. (**b**) Safe location.

**Figure 14:**
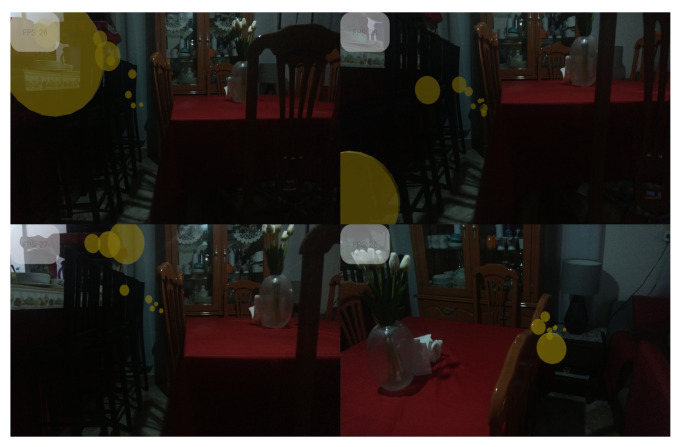
Virtual elements on the path to avoid obstacles in an unknown environment.

Once the systems display virtual elements, a user experiment describes the performance employing a virtual coin route to guide the users to evaluate the interaction with the physical world. Figure 13a describes an unsafe distance between barriers that could result in an accident. Figure 13b depicts the safe location for virtual objects to avoid traversing the obstruction. Real-time deployment of virtual elements in a physical environment is depicted in Figure 14. The position for each coin that composes a virtual path prevents potential collisions.

According to [46], there are three important factors to consider when evaluating the performance of a 3D application: the participation of representative users, the environment of the evaluation, and the sorts of findings generated. The evaluation is based on the user’s interaction with the proposed system. Eight users conducted four system evaluations in this investigation and monitored user behavior. Table 4 details the number of potential collisions between each user and the obstruction.

Over time, each user adjusts to the system based on the outcomes of their user experience. Once the user has used the system for the first time, the user typically mistrusts the location of the virtual elements and maintains a keen awareness of the real world. In other words, the user looks over the mobile device out of concern for a potential collision. The experiment demonstrates how individuals adapted to a system with a high number of potential collisions; each user had at least three collisions, although in some cases, their fear of colliding through the system decreased to zero or one. Consequently, the user focuses on the device’s screen, reducing the fear of colliding with an obstruction, and the system enhances the safety of traveling in a controlled environment.

Therefore, this study presents an alternate technique for providing a path generator for displaying virtual elements with minimal information, which can be implemented in uncertain environments. Likewise, a novel alternative has been established for deriving a general solution from restricted information to avoid specific data information in the environment.

## 5. Conclusions and Future Work

This research presents a way to resolve the problem of building unpaired datasets for indoor exploration using augmented reality applications on limited devices using the transfer style. This proposal provides an alternative to expanding the limits of specific datasets to specific solutions. Therefore, the transfer style enables connection to unknown domains based on a known environment, which contains the generated paths, and the GAN approximates a potential solution according to sharing features. Furthermore, it is crucial to note that this method delivers a real-time understanding of the physical world because the virtual elements are displayed in a safe location. Due to this behavior, this approach expands the potential implementations of GANs.

Although technologies such as Arcore and Kinect sensors have been utilized to evaluate the possibility of collisions, these technologies require the online execution of a path planner generator. Consequently, the development and implementation time is costly, but the precision is superior. However, this proposal helps reduce the number of external sensors and avoids knowing the environment in advance, two of the most significant limitations of the technologies, as mentioned earlier. In addition, the experience is real-time, and the most innovative aspect is the execution on constrained devices such as smartphones.

According to user experience, the user improves with time while learning to concentrate on the immersive experience without relying on prior knowledge of the environment. However, additional concerns have emerged, such as the minimum number of available samples required to ensure optimal behavior and how this type of solution compares to physical sensors. On the other hand, the concept of meta-learning could supplement this work by enabling the creation of a generic solution for scenarios that share similar features without the need for several samples of the same objects.

The objective of future work will be to address the challenges caused by the introduced work and to supplement this proposal with other methods, such as meta-learning. Meta-learning might be applied to fine-tune potential global solutions whose characteristics are adequate to facilitate the current disadvantages of the present effort.

## Figures and Tables

**Table 1:** Description of each domain employing average and dominate color.

	Average Color		Dominate Color	
	Mean	STD	Mean	STD
Known environment	110.9818, 110.6834,125.3244	11.1791,11.2014, 6.3755	114.2768, 171.9477, 170.8425	18.5680, 12.0359, 8.7037
Transfer features	84.0189, 84.2476, 83.5052	5.0146, 5.4588, 5.5117	11.9112, 12.0532, 11.9745	3.3174, 3.4040, 3.5008
	119.9242, 125.1150, 123.8134	12.2781, 6.5528, 7.4241	179.4007, 214.4179, 193.91524	8.8878, 7.6791, 13.3808
	128.9802, 143.6930, 140.1511	13.6080, 10.2819, 9.3439	174.8663, 208.5393, 175.3898	14.4137, 9.6241, 11.1316
Normal features	120.8573, 120.9270, 120.8453	12.9963, 12.9853, 12.9868	83.6730, 83.6655, 83.6190	16.7216, 16.6732, 16.5330
	110.5359, 97.0221, 112.8038	24.3627, 28.4992, 6.5405	109.7473, 97.8742, 196.9221	27.4893, 28.5040, 14.3613
	163.2307, 149.6583, 184.5755	4.9271, 6.2114, 2.9584	231.9854, 207.9542, 217.7950	4.0251, 4.4566, 6.5562

**Table 2:** Features description for each unknown domain.

Domain	Transfer Features		Normal Features	
	**Centroid**	**Distance**	**Centroid**	**Distance**
Known	0.1565, 0.3084, −0.6504	-	0.1565, 0.3084, −0.6504	-
Unknown A	0.1741, 0.3108, −0.5918	0.0612	0.1934, 0.3004, −0.3697	0.2832
Unknown B	0.1549, 0.3215, −0.6529	0.0134	0.1553, 0.3260, −0.5249	0.1267
Unknown C	0.1365, 0.3254, −0.6811	0.0403	0.1410, 0.3047, −0.5773	0.0748

**Table 3:** Performance of each model to generate a path with 50 evaluations, where (↓) Low is better and ↑ Up is better.

Environment	Model	Accuracy Euclidean Distance (Mean-Sdt) (↓)	Accuracy Manhattan Distance (Mean-Sdt) (↓)	Accuracy Cosine Similarity (Mean-Sdt) (↑)	Free Collision Coefficient (↑)
A	Autoencoder	2.4481 ± 0.7458	4.9868 ± 1.6354	0.7896 ± 0.0896	0.78
	HC	1.3789 ± 0.3489	3.4579 ± 0.8978	0.8928 ± 0.0480	0.88
	TL-Autoencoder	7.7589 ± 1.7895	11.4587 ± 2.4756	0.5478 ± 0.0789	0.52
	TL-HC	2.1458 ± 0.7458	3.2458 ± 0.8756	0.8878 ± 0.0478	0.86
	ARcore	0.8253	1.3268	0.8647	0.92
	Kinect	0.5146	0.9984	0.8964	0.96
B	Autoencoder	3.4656 ± 1.4183	5.0145 ± 0.9315	0.7265 ± 0.1018	0.79
	HC	1.8425 ± 0.5792	2.9854 ± 0.7419	0.8472 ± 0.0478	0.86
	TL-Autoencoder	5.2947 ± 1.2415	9.6542 ± 1.7892	0.6217 ± 0.1479	0.59
	TL-HC	1.9475 ± 0.4531	4.0154 ± 0.6548	0.8934 ± 0.1256	0.89
	ARcore	0.9205	2.0268	0.7908	0.88
	Kinect	0.7251	1.1356	0.8955	0.94
C	Autoencoder	2.1946 ± 0.6026	3.7146 ± 0.7987	0.8476 ± 0.1796	0.82
	HC	0.9987 ± 1.0147	2.5892 ± 0.9735	0.8956 ± 0.0145	0.90
	TL-Autoencoder	5.8624 ± 1.9752	10.2305 ± 1.0174	0.6204 ± 0.0497	0.59
	TL-HC	2.2580 ± 0.0325	4.0563 ± 0.6520	0.8942 ± 0.0041	0.88
	ARcore	0.9527	1.159	0.8872	0.96
	Kinect	0.8745	1.3746	0.9004	0.96
D	Autoencoder	1.9524 ± 0.8456	3.2168 ± 0.5428	0.8128 ± 0.1558	0.84
	HC	2.0415 ± 0.5478	4.0127 ± 0.7812	0.8872 ± 0.0147	0.92
	TL-Autoencoder	4.4527 ± 1.2305	9.4762 ± 1.9856	0.5856 ± 0.0586	0.62
	TL-HC	2.7586 ± 0.2569	5.9682 ± 0.4368	0.9028 ± 0.1868	0.88
	ARcore	0.8898	1.5743	0.9246	0.98
	Kinect	0.9975	1.7104	0.9165	0.96

**Table 4:** Description of the performance with users to avoid possible collisions in a physical environment.

Experiment	User 1	User 2	User 3	User 4	User 5	User 6	User 7	User 8
1	4	6	5	4	4	3	7	5
2	4	3	4	4	3	1	4	3
3	2	1	2	1	1	0	2	1
4	0	1	1	0	0	0	0	1

## Data Availability

Not applicable.
